# Designing a Gait Recognition Algorithm for Older Adults Using Mobility Aids: Prospective Cohort Study

**DOI:** 10.2196/68669

**Published:** 2025-11-10

**Authors:** Samantha Jeane Ray, Jung In Koh, Amanda Mae Liberty, Tracy Anne Hammond, Paula Kay Shireman

**Affiliations:** 1 College of Medicine, Texas A&M University, MREB 2 8447 John Sharp Parkway, Bryan, TX, 77807, United States, 1 9364638530; 2 College of Engineering, Texas A&M University, College Station, TX, United States

**Keywords:** gait recognition, walking recognition, older adults, wearable technology, activities of daily living

## Abstract

**Background:**

Maintaining mobility is important for older adults to retain independence and reduce fall risk. Wearable technology such as fitness trackers and smartwatches can track physical activity. Unfortunately, gait recognition algorithms are often calibrated using younger adults and are not accurate for older adults, especially when using mobility aids.

**Objective:**

Our goal was to develop a gait recognition algorithm capable of detecting the walking patterns of older adults that is robust to using mobility aids. Wrist-worn wearable devices were used to maximize the ubiquity of the approach.

**Methods:**

We collected walking and other daily activity data on 17 independent older adults to develop a gait recognition algorithm. Ten participants used mobility aids (ie, 5 cane users, 4 rollator users, 1 walker user). We calibrated a heuristic-based “one-size-fits-most” algorithm leveraging the harmonic patterns associated with walking to recognize the walking patterns of our cohort. This algorithm is computationally lightweight and relies only on accelerometer data. We used hyperparameter tuning using a Parzen tree estimator to find the optimal parameters in a leave-one-subject-out fashion.

**Results:**

The calibration process was required for this algorithm to detect walking. The signal amplitude threshold lowered from 0.3g to 0.1g to detect the more subtle walking patterns of older adults. The walking frequency range widened from [1.4Hz, 2.3Hz] to [0.8Hz, 2.8Hz], showing that older adults walk more slowly. The ratio for superharmonics increased from 1.4 to 77.11. Analyzing the false positive rate for the other daily activity classes implies that these superharmonics are artifacts of back-and-forth arm motions that characterize walking in our collected data. Additionally, we report the performance metrics of sensitivity, specificity, and *F_1_*-score to evaluate our algorithm. Sensitivity increased from 0.11 to 0.73; *F*_*1*_-score increased from 0.15 to 0.73; and specificity decreased from 0.99 to 0.75.

**Conclusions:**

This study successfully recognized the walking patterns of older adults with or without mobility aids. The performance metrics show that this algorithm has promise for being used to monitor physical activity. This approach is computationally lightweight and explainable. Our calibration approach can be adopted to tune to new populations and has a low barrier to entry for adopting a new technology due to the sole reliance on accelerometer data which is a standard sensor in wearable devices. The most noteworthy parameter adjustment is the ratio for superharmonics. Low values for the subharmonic and superharmonic ratios cause the algorithm not to detect walking in our older adult data. We validated the algorithm on ten mobility aid users. A larger study with more participants using mobility aids is necessary to conduct a deeper analysis on what parameters work best for this population. Future work includes validating the algorithm’s ability to estimate step counts and measure physical activity in real-world settings.

## Introduction

Detecting changes in lower body function is crucial for preventing falls and maintaining mobility and quality of life [1-3]. Falls can be life-threatening for older adults; approximately one-third of individuals over the age of 65 experience a fall each year [4] and 55.8% of accidental deaths are due to falls [5]. Moreover, impairments in lower body function increase the risk of falls [6,7] and can also lead to more severe outcomes, such as hospitalization [8,9], loss of independence [10], and fear of falling, which further restricts physical activity [11-14]. Many individuals fail to recognize the deterioration in their health until after experiencing a fall. Early signs of health decline in older adults are often overlooked until hospitalization occurs, thereby missing critical opportunities for early intervention [15]. In addition, many older individuals fail to acknowledge the severity of their health decline until after a significant event, such as a fall [16,17]. Thus, early detection of lower body function deterioration allows for personalized interventions, such as physical therapy or the use of mobility aids, which can help mitigate these risks [18-20]. Unfortunately, technologies to monitor lower body function to detect subtle changes in older adults are not available, especially when mobility aids are used.

Wearable sensors have emerged as a widely recognized tool for monitoring physical activity in real time, offering essential data on health and mobility [21,22]. The market currently offers numerous options for activity trackers, including wrist-worn fitness trackers like Fitbit (Google LLC) [23] and smartwatches such as Apple Watch (Apple Inc.) [24] and Samsung Galaxy Watch (Samsung Electronics Co., Ltd.) [25]. These devices measure various metrics such as step counts, heart rate, walking speed, cadence, and posture. Wearable technology can assist in the early detection of mobility issues and predict fall risks, especially through gait pattern and walking dynamic analysis [4,26]. Moreover, in clinical settings, wearable sensors are increasingly used to assess rehabilitation outcomes [27-29], remotely monitor patients [30-32], and manage chronic conditions [33-36], thereby providing continuous data outside traditional health care environments.

However, these algorithms have not been optimized for older adults who rely on walking aids. The altered biomechanics caused by mobility aids such as rollators disrupt natural gait patterns which can impact the accuracy of gait detection. Specifically, older adults tend to walk more slowly and with shorter strides, creating challenges to accurately detect subtle movements for these algorithms developed using younger populations [37]. Furthermore, using mobility aids changes how people walk, especially from the perspective of wrist-worn sensors. For example, arm swings may be absent or reduced and patterns indicating footfalls may be sharpened or dampened. These disruptions in gait patterns frequently cause wearable sensors to misinterpret or underestimate mobility levels, resulting in unreliable analysis for the collected data [38,39]. Consequently, this limits the effectiveness of wearable devices in monitoring activity levels or assessing fall risks in individuals using mobility aids. Although some research has explored this issue [37,40,41], further studies are needed to fully understand the underlying dynamics and address these limitations.

This work focuses on the development and evaluation of a gait recognition algorithm that is robust to the usage of mobility aids. We calibrate a “one-size-fits-most” walking recognition algorithm [42] to be sensitive to the walking patterns of older adults with or without the use of mobility aids.

## Methods

### Study Design

We conducted a prospective cohort study with 17 older adults living in independent living. The study design focuses on capturing labeled samples of walking behaviors and other activities of daily living (ADLs) in the participant’s normal environment. The other ADLs include brushing teeth, combing hair, drinking, eating, taking medication, transitions, and washing hands. Transitions include sit-to-stand, stand-to-sit, sit-to-lie, lie-to-sit, sitting still, laying still, bending down, and straightening up. Participants were allowed to opt out of performing any activity at their discretion.

Motion data from these activities was collected using a wrist-worn sensor on the dominant hand. This study uses Samsung Galaxy Watch 6 smartwatches (Samsung Electonics Co., Ltd.) [25]. Accelerometer data was sampled at 50Hz using a custom Android application.

Annotation used a seminaturalistic style: participants were given specific activities and were allowed to perform the activities as they would naturally. Study personnel used a mobile phone app to label the occurrence of each event. This labeling app captured exact timestamps that activities started and stopped; these labels were later synchronized with the smartwatch accelerometer data.

Annotation sessions took place in the participant’s apartment or the gym area of the retirement community. Participants were instructed to follow their normal routines for brushing teeth, combing hair, taking medication, and washing hands. Participants were asked to take at least 3 sips for drinking and 3 bites for eating. Likewise, participants were asked to repeat transition behaviors 3 times. Walking events lasted several minutes and involved walking from one location to another within the retirement community (eg, walking from the gym back to the participant’s apartment). Participants were not given any time minimums while performing the activities.

### Ethical Considerations

The study protocol was approved by the Texas A&M University Institutional Review Board (IRB2020-1271D). All participants provided informed consent. The study design follows standard procedures for human subjects research to minimize risk to participants. Participants were able to withdraw from the study at any time with no penalty. Data were deidentified during analysis. The study provided no compensation.

### Recruitment

Recruitment took place at two retirement communities in the southwestern United States. The research team advertised by giving presentations about the study at each location with permission from each location’s administrative team. Interested residents contacted the research team to join the study.

### Algorithm Design

The “one-size-fits-most” algorithm leverages the inherent physical characteristics of taking steps to detect bouts of walking. Walking is defined as sequences of strides (ie, two consecutive steps) and has attributes such as intensity and periodicity. By this definition, walking can be distinguished from other daily activities by searching for windows of sensor data that satisfy these characteristics.

This algorithm searches for 1-second windows, where the accelerometer vector magnitude is above a given threshold, A, in meters per second-squared. Data is resampled to 10Hz to minimize the data fidelity requirements. Additionally, lower sampling rates reduce the risk of capturing confounding patterns as walking is not a fine-grained activity.


(1) $$\alpha {\;}^{*}\;p_{w}>\;p_{min}$$

(2) $$\beta {\;}^{*}\;p_{w}>\;p_{max}$$

Valid windows are transformed into the frequency domain using a continuous wavelet transform (CWT) to produce C(f, 𝜏), the decomposition of the signal into scaled, time-shifted wavelets where f is the frequency and τ is the time step. These segments are checked against a series of conditions regarding their peak frequencies. The overall peak is expected to be within the frequency range f_w_=[f_min_, f_max_]. Three peaks within C(f, 𝜏) are detected: the subharmonic peak p_min_, the walking range peak p_w_, and the superharmonic peak p_max_. The presence of superharmonics and subharmonics are allowed if the peaks satisfy Equations (1) and (2). *α* and *β* are parameters that control the maximum ratios of the peak magnitudes.

These segments are marked as candidates for walking. The final step checks if the sequences of candidate segments are longer than the minimum time threshold, T. This step removes short bursts of activity that are unlikely to be an actual bout of walking, improving the algorithm specificity.

The values for A, *α*, *β*, f_min_, f_max_, and T come from analyzing walking from 20 existing datasets with a total of 1240 healthy participants, as previously reported [42]. Fifteen of the datasets focus solely on mobility behaviors; the other 5 include other daily activities. Age and sex reporting varied by dataset. Age was reported for 745 participants with range 15‐75 years (mean 28.6, SD 12). Sex was reported for 901 participants and 72% (n=649) were male. There are two sets of parameters depending on the location of the sensor. For wrist-worn sensors, the default values are A=0.3g, *α*=31.7, *β*=1.4, f_min_=1.4Hz, f_max_=2.3Hz, T=6 seconds [42].

We calibrate these parameters to reflect the walking patterns of older adults. We conduct hyperparameter tuning and use a leave-one-subject-out (LOSO) to explore multiple views of the data. We use a tree-structured Parzen estimator to explore the parameter space. A had the potential range of 0.05g to 0.5g. *α* and *β* had the range of 0 to 100. f_min_ varied from 0.6Hz to 1.5Hz; f_max_ varied from 2Hz to 3Hz. T had the range of 1 to 10 seconds. We used the hyperopt [43] Python package and 500 trials per fold of the LOSO evaluation. *F_1_*-score was used as the optimization metric to capture both sensitivity and precision. The final value of each parameter is determined by averaging across the folds and rounding to one decimal for all parameters except time, which is rounded to a whole number.


(3) sensitivity=TP / (TP+FN)

(4) specificity=TN / (TN+FP)

(5) $$F1-score=2^{*}\;TP\;/\;\left(2^{*}\;TP+FN+FP\right)$$

The algorithm estimates cadence in terms of steps per second. This output is converted to walking versus not-walking annotations by labeling windows with a non-zero number of steps as walking. Algorithm performance is measured through sensitivity (Equation 3), specificity (Equation 4), and the *F_1_*-score (Equation 5). Correctly detected seconds of walking are true positives (TP) and missed seconds are false negatives (FN). Conversely, correct non-walking seconds are true negatives (TN) and the corresponding errors are false positives (FP).

## Results

### Participants

Table 1 summarizes the demographic information of the recruited participants. All the participants were ambulatory. Some used mobility aids in their daily lives. One participant used a walker; their data is grouped with the rollator-users because they used the walker like it was a rollator .

**Table 1.** Participant demographic information organized by mobility aid usage.

Variables	All (N=17)	No aid (n=7)	Cane (n=5)	Rollator/Walker (n=5)
Age (years), n				
70‐79	5	1	2	2
80‐89	10	5	3	2
≥90	2	1	0	1
Female, n	12	5	2	5
Right hand dominant, n	16	6	5	5

### Parameter Tuning

The parameters from each fold of the LOSO evaluation are averaged to produce the calibrated parameters. The main algorithm parameters needed adjustment for older adults. The threshold A lowered from 0.3g to 0.1g. The parameters *α* and *β* increased from 31.7 to 65.4 and 1.4 to 77.1, respectively. The expected frequency range of walking expanded from [1.4Hz, 2.3Hz] to [0.8Hz, 2.8Hz]. The minimum time requirement increased from 6 to 10 seconds. The summary of these parameters is given in Table 2.

**Table 2.** Parameters for characterizing gait by algorithm version.

Parameters	Amplitude (A)	*α*	*β*	Minimum frequency (f_min_)	Maximum frequency (f_max_)	Time (T)
Default [42]	0.3	31.7	1.4	1.4	2.3	6
Calibrated	0.1	65.4	77.1	0.8	2.8	10

### Recognition Performance

We provide sensitivity, specificity, and *F_1_*-score metrics using the default parameters, the average performance using the parameters within the LOSO folds, and using the calibrated parameters (Table 3). The average values and the ranges are reported per metric. The default and LOSO rows are participant-independent results; the calibrated row shows the performance using the average parameters from hyperparameter tuning (Table 2).

**Table 3.** Algorithm performance of walking against ADLs^a^ organized by mobility aid usage.

Metrics and parameters	Overall	No aid	Cane	Rollator
Sensitivity (range)				
Default	0.11 (0.00‐0.72)	0.22 (0.01‐0.72)	0.04 (0.01‐0.09)	0.01 (0.00‐0.02)
LOSO^b^	0.74 (0.10‐1.00)	0.72 (0.10‐1.00)	0.67 (0.45‐0.95)	0.85 (0.75‐0.96)
Calibrated	0.73 (0.10‐1.00)	0.72 (0.10‐1.00)	0.64 (0.43‐0.95)	0.84 (0.74‐0.93)
Specificity (range)				
Default	0.99 (0.97‐1.00)	0.99 (0.97‐1.00)	0.99 (0.98‐1.00)	1.00 (0.99‐1.00)
LOSO	0.73 (0.20‐0.97)	0.68 (0.20‐0.88)	0.78 (0.56‐0.97)	0.76 (0.63‐0.84)
Calibrated	0.75 (0.32‐0.98)	0.71 (0.32‐0.90)	0.80 (0.56‐0.98)	0.76 (0.63‐0.86)
*F*_*1*_-score (range)				
Default	0.15 (0.00‐0.84)	0.31 (0.02‐0.84)	0.07 (0.01‐0.17)	0.01 (0.00‐0.05)
LOSO	0.73 (0.07‐0.94)	0.64 (0.07‐0.87)	0.71 (0.52‐0.83)	0.87 (0.74‐0.94)
Calibrated	0.73 (0.08‐0.92)	0.65 (0.08‐0.89)	0.69 (0.52‐0.85)	0.87 (0.75‐0.92)

a ADLs: activities of daily living.

b LOSO: leave-one-subject-out.

The default parameters have high specificity at 0.99 but poor sensitivity at 0.11. The resulting *F_1_*-score is low at 0.15. Participants who used no mobility aid had better performance than those who used them. Walking detection for cane users was slightly higher than that for rollator users with *F_1_*-scores of 0.07 and 0.01, respectively.

The LOSO results have balanced sensitivity and specificity metrics with both at 0.73. Cane-users had the lowest sensitivity at 0.67; rollator-users had the highest sensitivity at 0.85. Participants with no aid had the lowest specificity at 0.68; cane-users had the highest specificity at 0.78, closely followed by rollator-users at 0.76. No-aid participants also had the lowest *F_1_*-score at 0.64; rollator-users had the highest at 0.87, and cane-users had a score of 0.71.

The calibrated results are comparable to the LOSO results. Most metrics are within 0.05 of the corresponding metrics using the LOSO parameters. Sensitivity is slightly lower and specificity is slightly higher. *F_1_*-score is slightly better for no-aid participants, slightly worse for cane-users, and equal for rollator-users.

## Discussion

### Principal Results

All the parameters for the algorithm needed adjustment to detect walking for our prospective cohort. The threshold A was lowered to 0.1g, indicating that the steps taken by older adults are softer and more subtle. *α* and *β* are higher at 65.4 and 77.1 compared to 31.8 and 1.4, respectively. Subharmonics are dependent on the location of the wearable device, so the increase may just be a side effect from the smartwatch used in this study compared to the original work. Superharmonics can be caused by noise in the signal or environmental factors such as the walking surfaces. *β* is notably higher; this result implies that the patterns associated with walking are less distinct. The range of f_w_ is wider on both ends of the spectrum. f_min_ is lowered to 0.8Hz, aligning with the expectation that older adults walk slower. f_max_ increased to 2.8Hz which is surprising because this would imply that the participants walked faster than the average adult. This outcome may be connected to the more lenient values for A, *α*, and *β*. Strong peaks generally indicate footfalls. In this case, the footfall peaks may have been softer and surrounded by other peaks (ie, subharmonic and superharmonic patterns), making it appear as though the participant was walking faster.

The reasonable sensitivity of our algorithm suggests the potential for monitoring physical activity. The calibrated algorithm can be used to detect if an older adult has become sedentary. This algorithm is computationally lightweight as a rule-based algorithm that only relies on CWT for data processing; as such, it can be leveraged by standard wearable devices. Additionally, using data from only one arm is sufficient, suggesting that wearing a single arm sensor may be sufficient to detect clinically relevant changes.

The value of *β* has a notable impact on algorithm performance. We conducted a small ablation study to determine which parameters contributed to the higher false positive rates for some ADLs. Lower values for *β* reduced the false positive rate, but the true positive rate for the algorithm also decreased in turn, bringing performance back in line with the default parameters This outcome implies that the superharmonics in the walking samples are distinguishable characteristics. The superharmonic peaks likely are an artifact from the back-and-forth motions present in walking and several ADLs such as brushing teeth and washing hands.

### Comparison with Prior Work

This study addresses two common gaps in the gait recognition literature: inclusion of older adults as participants and inclusion of people using mobility aids. Furthermore, this study provides initial results into future work identified by the original work that proposed the “one-size-fits-most” algorithm [42].

### Limitations

A limitation of this study is the small sample size in terms of unique participants and dataset size in terms of seconds of sensor data. More data would improve the strength of the results. Future work should both increase the number of participants and the length of the data collection sessions. Additionally, future evaluations should take place in free-living environments where participants are allowed to behave naturally. These studies may focus on assessing the effectiveness of this algorithm as a sedentariness level tracker.

### Conclusion

This study successfully recognized the walking patterns of older adults with or without mobility aids. The performance metrics show that this algorithm has promise for being used to monitor physical activity. This approach is computationally lightweight and explainable because it only relies on the CWT computationally and the algorithm design is rule-based in nature. Our calibration approach can be adopted to tune to new populations and has a low barrier to entry for adopting a new technology due to the sole reliance on accelerometer data which is a standard sensor in wearable devices. The most noteworthy parameter adjustment is the ratio for superharmonics. The lower values for these ratios in the default parameters were a key reason why it could not detect walking in our older adult data. The calibrated parameters were more lenient to peaks outside the expected frequency range of walking. This outcome increases sensitivity while also increasing risk of false positives, hence the decrease in specificity.

We validated the algorithm on ten mobility aid users (ie, 5 cane, 4 rollator, and 1 walker). A larger study with more participants using mobility aids is necessary to conduct a deeper analysis on what parameters work best for this population. Future work includes validating the algorithm’s ability to estimate step counts and measure physical activity in real-world settings.

## Abbreviations

ADL: Activity of Daily Living
CWT: Continuous Wavelet Transform
FN: False Negative
FP: False Positive
LOSO: Leave-One-Subject-Out
TN: True Negative
TP: True Positive
